# CRISPR-Cas13-based amplification-free detection of three quarantine-significant sugarcane viruses

**DOI:** 10.1007/s00299-026-03994-4

**Published:** 2026-09-26

**Authors:** Joseph Lagner, Anna Paulson, Taylor Schulden, Bishwo Adhikari, Julian Koob, Christopher Vakulskas, Yiping Qi

**Affiliations:** 1 Department of Plant Science and Landscape Architecture, University of Maryland, College Park, MD 20740 USA; 2 Plant Protection and Quarantine, USDA Animal and Plant Health Inspection Service, Plant Germplasm Quarantine Program, Laurel, MD 20708 USA; 3 Integrated DNA Technologies, Coralville, IA 52241 USA; 4 Institute for Bioscience and Biotechnology Research, University of Maryland, Rockville, MD 20850 USA

**Keywords:** CRISPR-Cas13, LwaCas13a, RNA viruses, sugarcane viruses, amplifi cation-free, multiplexed detection

## Abstract

Crop losses from viral pathogens cost billions of dollars annually, and sugarcane is particularly vulnerable. Sugarcane is the most cultivated crop in the world by volume; therefore, significant yield losses result in large dollar losses for farmers and trade economies. In some cases, viruses contribute to more than half of the yield reduction. Three quarantine-significant pathogens—*Sugarcane mosaic virus* (ScMV), *Sugarcane streak mosaic virus* (ScSMV), and *Sugarcane yellow leaf virus* (ScYLV)— pose major threats to global sugarcane production because of their rapid spread, asymptomatic infections, and delayed symptom expression. Effective management of these pathogens requires robust, rapid, and accurate detection methods that can distinguish among these viruses, even at low titers. Here, we report the development of an amplification-free CRISPR-Cas13-based assay with a limit of detection (LOD) of ~1.5 pM. The assay allows for simultaneous screening of all three viruses directly from bulk RNA extracts of plant material. Our approach leverages LwaCas13a specificity to detect and differentiate viral targets in a single assay. Compared with conventional multi-step assays, this strategy significantly reduces time and contamination risk. Validation on naturally infected material demonstrated its sensitivity and reliability. This platform provides a powerful tool for early and accurate diagnosis of ScMV, ScSMV, and ScYLV, with strong potential for integration into sugarcane virus surveillance and management programs.

**Supplementary Information:**

The online version contains supplementary material available at https://doi.org/10.1007/s00299-026-03994-4.

## Introduction

**Fig. 1:**
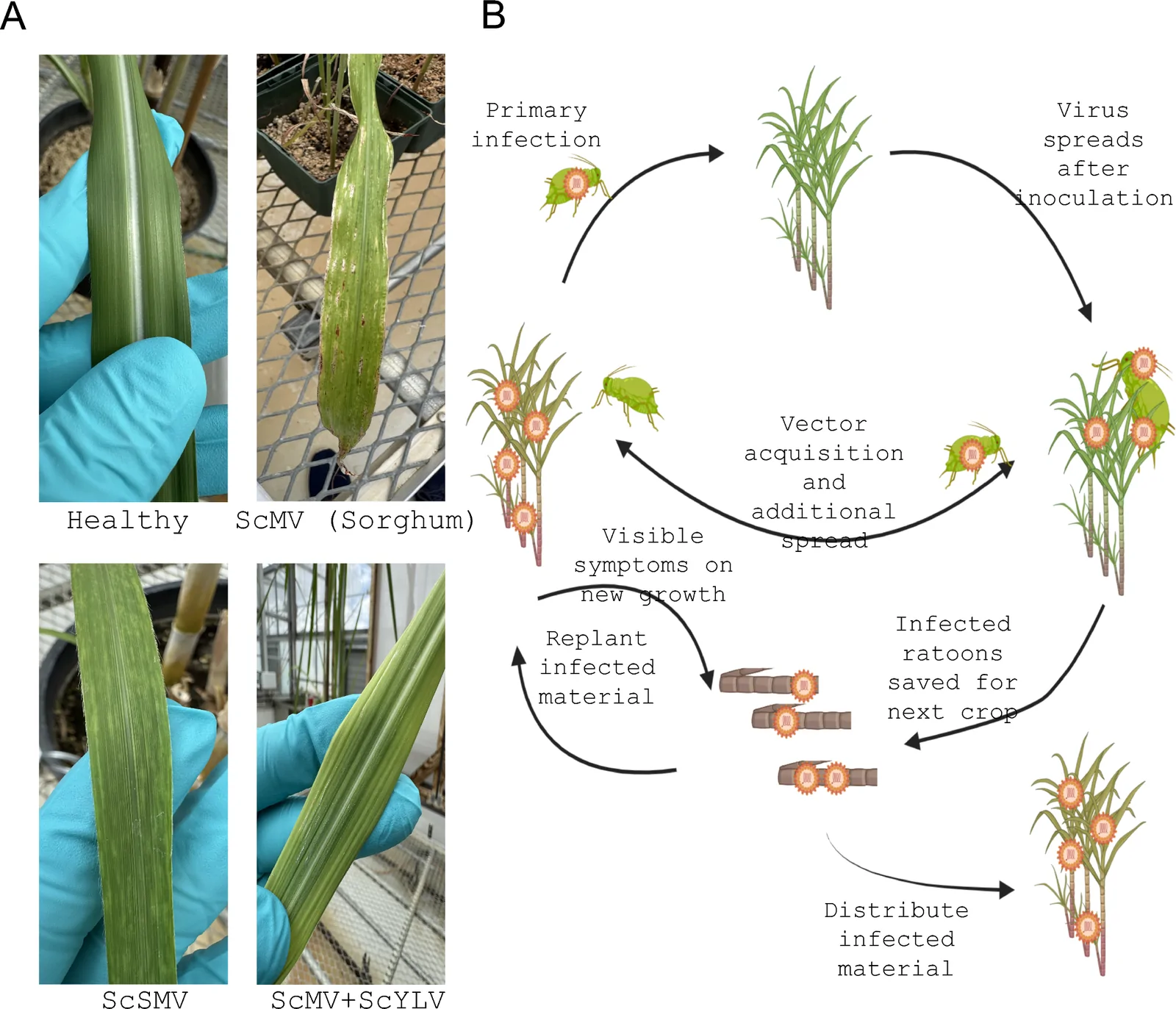
Virus infection symptoms and the modes of spread. (**A**) Symptoms of viral damage on sugarcane and sorghum hosts. Dual or multiple infections are also possible. (**B**) Diagram of how these target viruses are most commonly spread. Aphid insect vectors can transfer the pathogen onto healthy plants after feeding on infected material. The infected material may not show signs at first and may potentially be used for the following year's crop or be traded and sent to a new location. The diagram was generated with BioRender

Globally, sugarcane is the most cultivated crop, with an industry producing ~179 M metric tons annually and growing (OECD, 2025). This crop is an important staple that provides many uses beyond sweeteners, including biofuels, building materials, and medicinal compounds (Singh et al. 2015; Dinesh Babu et al. 2022). While global sugarcane production has been on the rise, some key producers have declined in production and found it challenging to produce the same or more yield (Feng et al. 2023; Headley et al. 2024, 2025). Factors range from climate change, farming practices, and shifts in global needs. However, a major factor that affects the yield of all cultivated crops is the presence of pathogens in growing operations (Kumar et al. 2018; Batz et al. 2021; Njeru et al. 2023). Sugarcane is no exception, with some fields reporting up to 80% yield loss, even in elite, supposedly pathogen-resistant cultivars (Holkar et al. 2020; Lu et al. 2021; Vamsi Krishna et al. 2023). Major contributors to these staggering losses are 3 particular viruses that have been classified as quarantine-significant: Sugarcane Mosaic Virus (ScMV), Sugarcane Streak Mosaic Virus (ScSMV), and Sugarcane Yellow Leaf Virus (ScYLV) (Viswanathan and Balamuralikrishnan 2005; Wu et al. 2012; ElSayed et al. 2015; Jones 2021). ScMV and ScSMV both belong to the *Potyviridae* family. These viruses are single-stranded, positive-sense RNA viruses with one open reading frame (ORF) (Shukla 1989; Chen et al. 2002; Xu et al. 2008). ScYLV is also an ssRNA virus, yet it contains 6 ORFs and belongs to the *Luteoviridae* family (ElSayed et al. 2015; Bagyalakshmi et al. 2019; Holkar et al. 2020). While these viruses are distinctly different, they often can be involved in co-infections (Fig. 1A). These viruses are most commonly vectored by aphids or vegetative propagation of infected materials (Lu et al. 2021) (Fig. 1B). Global commerce in sugarcane plant material has led to these viruses being reported in places that previously had not seen these high-interest pathogens (Mollov et al. 2016; Sorho et al. 2021; Xu et al. 2021). Sugarcane is not only agronomically relevant, but also economically as well, with these viruses impacting global and local economies that rely on the sugarcane trade (Vijai Singh 2003; Putra et al. 2014). It is imperative to develop strategies to safeguard this industry from any further loss in yield.

CRISPR has been a world-changing discovery that has completely reshaped how to perform molecular genetic manipulation (Malzahn et al. 2017; Cox et al. 2017; Anzalone et al. 2020). The impact of CRISPR cannot be overstated; however, there are other applications of CRISPR outside of genome editing. Certain families of CRISPR-Cas proteins exhibit unique behavior: they can change their nuclease activity after a successful complementary match to the CRISPR RNA (crRNA) is found (Yan et al. 2019; Swarts and Jinek 2019; Tong et al. 2021; Huang et al. 2022). Cas12 (type V) and Cas13 (type VI) are most commonly used for this activity. It has been demonstrated that these proteins’ non-discriminatory activity (*trans*-cleavage; collateral cleavage) after finding their complementary match can be harnessed to signal when this activity has been activated (Gootenberg et al. 2017, 2018; Kellner et al. 2019; Ackerman et al. 2020). This is usually done by incorporating a reporter system that creates a detectable signal when targeted by the trans-cleavage activity (D Samanta 2022; Yin et al. 2022; Rossetti et al. 2022; Shrikrishna et al. 2024). Some CRISPR-Cas proteins have even been applied for the detection of specific genetic targets, as well as non-nucleic materials (J Y Hu 2021; D Samanta 2022; M M Chen 2022; Su et al. 2023; Cao et al. 2025). This technology has made rapid progress in detecting pathogens and molecular targets, yet it has not replaced established detection technologies such as quantitative PCR (qPCR), high-throughput sequencing (HTS), and Enzyme-Linked Immunosorbent Assay (ELISA). This is partly because we trust these technologies, which have been repeatedly validated and optimized over many years (Gyllensten and Erlich 1988; Mead et al. 1991; Viswanathan et al. 2010; Malapi-Wight et al. 2021; Aydin et al. 2025). While CRISPR genome editing was adopted remarkably fast, CRISPR-based detection has been slower to gain traction. Many advancements have improved CRISPR-based detection, strengthening its detection capacity (Wheatley et al. 2021; Marqués et al. 2022; Wang et al. 2023; Li et al. 2023).

Recently, we developed a CRISPR-Cas12a-based system for amplification-free detection of DNA-based plant pathogens such as phytoplasma (Lagner et al. 2025). Here, we sought to develop a new CRISPR-13-based detection system to simultaneously detect and identify three RNA viral pathogens directly from extracted bulk RNA, amplification-free. The application of CRISPR-Cas13 from *Leptotrichia wadeim* (LwaCas13a) for pathogen detection was pioneered with the development of the SHERLOCK platform for detecting human pathogens (Gootenberg et al. 2018; Kellner et al. 2019). A pre-amplification step has been used in many other CRISPR-based detection assays, and while it does provide an added sensitivity, it is often at the cost of specificity and potential contamination between steps. Most CRISPR-based detection assays under development incorporate isothermal strategies like RPA or LAMP (Mahas et al. 2021; Javalkote et al. 2022; Chandrasekaran et al. 2022; Bhat et al. 2022). While earlier methods required pre-amplification, it was later shown that LwaCas13a could confer amplification-free detection of RNA viruses, such as SARS-CoV-2 (Shinoda et al. 2021). Using LwaCas13a, we sought to develop a detection assay that is sensitive, specific, and significantly faster than conventional diagnostic assays used to detect these viral pathogens in suspected infected plant material. We wanted our system to work directly on extracted RNA without a pre-amplification step, which has increased interest in CRISPR-based screening (Fozouni et al. 2021; Shinoda et al. 2021; Lagner et al. 2025; Zhang et al. 2025). Hence, the assay we aimed to develop would be significantly faster than conventional diagnostic methods.

## Materials and methods

### Designing target sites

Sequence data of the three viruses were obtained from USDA-APHIS, PGQP (Laurel, MD, USA). Sequence alignments were created using Geneious Prime software with the MUSCLE alignment feature. The ‘PPP’ algorithm, which is the preset standard for MUSCLE alignment, was used. Based on these alignments, potential ORFs and functional protein domains were mapped using other features in Geneious. Initially, the Primer function of Geneious was used to map primers onto our sequences using parameters to match the spacer length for LwaCas13a (28 bp) with 100% similarity. Each potential site was manually picked and then screened on NCBI-BLAST to avoid potential non-specific effects. A potential site was disregarded if there were any signs that the site might be able to detect a similar species, plant genetic material, or another species that might be in close association with sugarcane production. Five sites were selected for each virus for experimental testing (Suppl. Table 1). Next, the reverse complement sequences of these target sites were added to the 3’ end of the direct repeat sequence for LwaCas13a (GACUACCCCAAAAACGAAGGGGACUAAAAC) to create the desired crRNA sequence. These ssRNA sequences were then ordered from IDT’s ‘CRISPR Custom Guide RNAs’ service (Integrated DNA Technologies, Coralville, IA, USA).

### Synthesis of ssRNA as a pseudo-virus for establishing the CRISPR assay

The pseudo-virus sequences containing the target sites were created by blending the five 28-nt target sequences of the virus into a synthetic DNA gBlock (synthesized by IDT) of 700 bp length with scrambled DNA sequences in between using an online “Random DNA Generator” service (https://faculty.ucr.edu/~mmaduro/random.htm). Within each gBlock, the intended target sequences were all on the same strand, driven by a T7 promoter, to accommodate in vitro transcription (IVT), which was done using the MegaScript™ T7 Transcription Kit Plus (Invitrogen, Waltham, MA, USA). The ssRNA synthesis was achieved by following the instructions provided with the kit, creating ssRNAs as target materials for subsequent testing. Each run was then checked by gel electrophoresis for the quality of synthesis and the correct size of the amplicon. The kit instructions were followed to make a 1:300 dilution to ensure reliable results. Samples were stored in 4 °C fridge, on ice, if used on the same day. Otherwise, they were stored in a −80 °C freezer for longer-term storage.

### Preparation of LwaCas13a protein

The wildtype CRISPR-LwaCas13a system was used in our detection assay. The LwaCas13a protein was obtained from IDT as part of the collaboration. The protein purification process follows a similar protocol that has been previously reported for Cas12a (Lagner et al. 2025). The protein was stored in aliquots in a −80 °C freezer until used for testing.

### Establishment of LwaCas13a assay for sugarcane viruses

An essential component for most CRISPR-based detection assays is fluorescent reporter oligonucleotides that provide an increase in fluorescence when detection has been achieved. In this study, the ssRNA oligo reporter was configured with a FAM fluorophore on one end and a fluorescent quencher on the other end (/56-FAM/rArArArArArUrUrUrUrU/3IAbRQSp/), and this reporter was ordered from IDT. When bound together on the same oligo, the quencher will absorb the fluorescence given off by the FAM molecule due to fluorescence resonance energy transfer (FRET). After LwaCas13a/crRNA has successfully found its target site, a conformational change of the RNP will unleash its non-specific activity to cleave any ssRNA material it encounters, causing a detectable increase in fluorescence when the FAM fluorophore dissociates from the attached quencher. The primary microplate reader used in this study was the Tecan Spark Multimode Reader (Tecan Trading AG, Switzerland). Run parameters were set to screen for FAM with recommended EX/EM wavelengths (483/20 nm; 525/20 nm). The protocol was programmed to run for 2 h at 37 °C with a brief shake every 2 min and then record fluorescence of each well on the plate. The plate that was used for these experiments was the Nunc 96-well optical bottom plate with a clear, removable top. A master mix was prepared for each experimental run, with a final concentration of 50 nM of LwaCas13a, 62.5 nM of crRNA, 50 nM of reporter oligos, and 1× NEB CutSmart Buffer (New England Biolabs, Ipswich, MA, USA) in a final reaction volume of 50 µL per testing well. In the 50 µL volume, 45 µL was the master mix and the other 5 µL was the IVT-synthesized ssRNA. The prepared ssRNA was log-diluted for testing to establish the limit of detection (LOD). Upon preparation, the sample plate was quickly loaded to the Tecan Spark machine to begin the run. After the run was completed, the raw reads from the machine were analyzed with GraphPad Prism software to generate figures presented in this paper. A positive detection was considered to occur when the mean of three replicates produced higher Relative Fluorescence Units (RFU) detected from the non-template control (NTC), which only contained water as ‘template’. A sample that is detecting a sample will also have an upward trend, while NTC wells should be stagnant from beginning to end.

### Extraction of RNA from sugarcane and sorghum plant materials

RNA extraction from sugarcane and sorghum plant materials was conducted at the USDA-APHIS, PGQP lab. This laboratory had infected plants and a proper biocontainment level for work with these quarantine-significant viruses. The leaf of each plant to test was photographed prior to sampling, before being used for the extraction protocol. Sorghum tissue was collected along with sugarcane because it has been characterized as an alternative host for ScMV. ‘Rio’ and ‘Z1536’ are extractions from the same plant individual, but these extractions were taken at two different times spaced about a month apart from each other. Since viral titer can fluctuate throughout the year and these extractions were done at different times, these samples will be considered as distinctly different. All samples were then lysed using a rolling macerator machine. RNA was then extracted following the instructions from the Qiagen RNeasy Plant Pro Kit (QIAGEN, Aarhus, Denmark). Concentration of bulk RNA extraction was quantified using a Qubit 4 Fluorometer. Samples were stored in 4 °C fridge if used on the same day, otherwise they were stored in a − 80 °C freezer for longer-term storage.

### LwaCas13a detection with RNA from plant tissues

For the LwaCas13a-based detection, each sample was tested using 4 wells on a microplate: one containing a multiplex detection using all crRNAs, and then one well for each of the viruses (ScMV, ScSMV, and ScYLV). This strategy was designed to test for any of the viruses that might be present in the well by multiplexing all of the crRNAs for each virus, and then each virus individually in a single-plex manner with each additional well. The setup is similar to the cross-reactivity test of our crRNA target sites. Samples were loaded into each testing well of the plate, mixed with the CRISPR-Cas13 reagents, and then analyzed in a fluorescent microplate reader. The extracted bulk RNA material was used to perform the assay with no further dilutions. The same reaction setup was used, including LwaCas13a protein (50 nM), crRNA (62.5 nM), 50 nM of reporter oligos, and 1× NEB CutSmart Buffer. In this case, a BMG Labtech VANTAstar machine (Cary, NC, USA) was used as a plate reader for signal capture. A run protocol was made following similar parameters on the Tecan Spark as closely as possible. It was found that using 500 nM (10× higher than assay establishment on the Tecan Spark) of reporter oligo worked better in test runs after making some optimization adjustments for this machine. Run data was saved and then analyzed in a similar manner using GraphPad Prism software. Notably, we background-subtracted from the Blank/NTC wells’ average RFU values over the course of the run. This was necessary when trying to reduce background noise. Our threshold was any average RFU value above one standard deviation of the highest ‘Negative Control’ (healthy plant material) value.

### Conventional RT-PCR detection assay

There were five individual assays performed to test for all the viruses that were used on each plant sample, for benchmarking our CRISPR-Cas13-based detection method. Proprietary protocols and primer sets designed by Agdia (Agdia, Inc., Elkhart, IN, USA) were used with contracted collaboration with USDA-APHIS, PGQP. Any questions or interest in using these protocols/primers should be directed to Agdia to seek their assistance (https://www.agdia.com/testing-services/potyvirus-group-test).

## Results

### Designing Target Sites for CRISPR-LwaCas13a

**Fig. 2:**
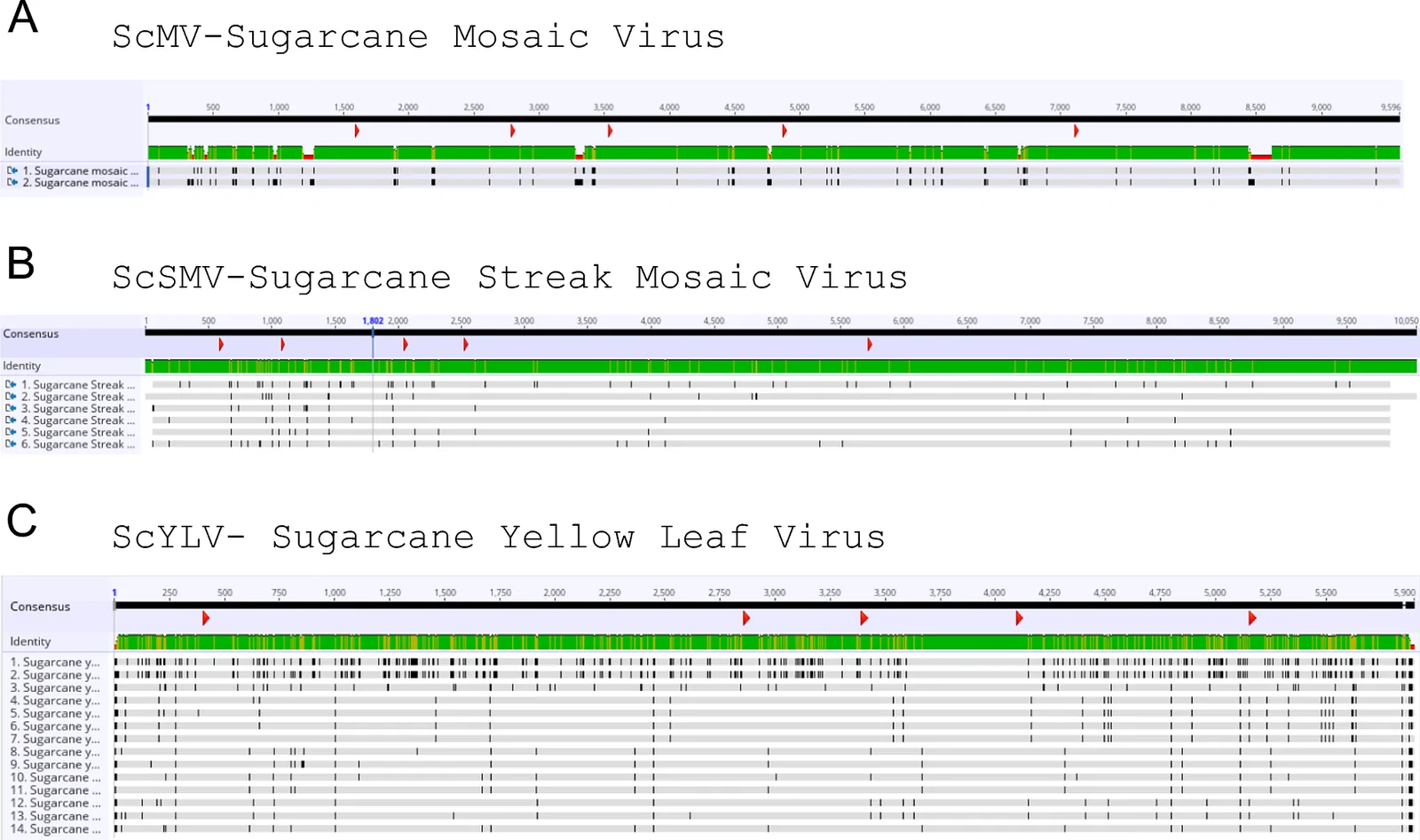
Alignment of the sugarcane viruses of interest. This alignment was generated using Geneious Prime software and the MUSCLE alignment function (Green represents a 100% match; Yellow represents 30–99% match; Red represents ≤30% match). We used the primer mapping function to identify suggested crRNA target sites, shown by the blue arrows. Red arrows are the target sites we selected for our detection testing

CRISPR-Cas13 is a powerful nucleic acid manipulation tool that interacts with genetic material, guided by its crRNA. To activate CRISPR activity, the first step is to design specific crRNAs for the target viruses. We received sequencing data from the USDA-APHIS laboratory for our three sugarcane viruses of interest. From these sequences, crRNA sequences were designed for each of the viruses. Five potential target sites were designed in silico for each virus (Fig. 2A-C). These target sites were then screened on NCBI-BLAST to ensure that there were no problematic off-targeting that could occur with our intended crRNA target sites.

### Establishing CRISPR-Cas13-based Detection for ScMV, ScSMV, and ScYLV

**Fig. 3:**
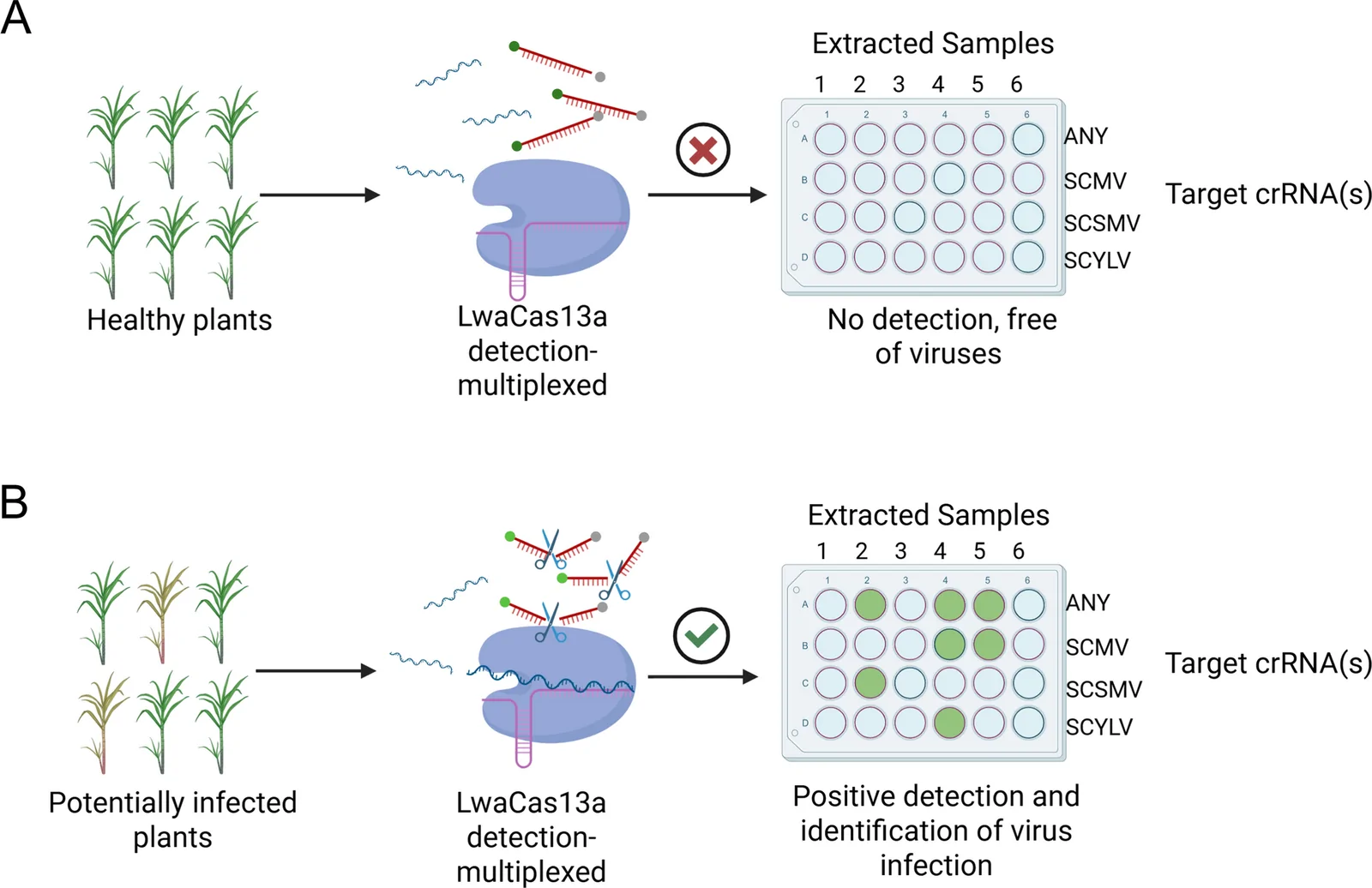
Our proposed assay for the simultaneous detection and genotyping of three quarantine-significant sugarcane viruses (ScMV, ScSMV, ScYLV). (**A**) Depiction of negative results from samples sourced from healthy plants. (**B**) Depiction of positive results that different viruses may be detected in infected plants. Multiplex crRNAs (ANY) and simplex crRNAs will facilitate efficient identification of plant materials infected with one or more viruses. Diagrams were generated with BioRender

**Fig. 4:**
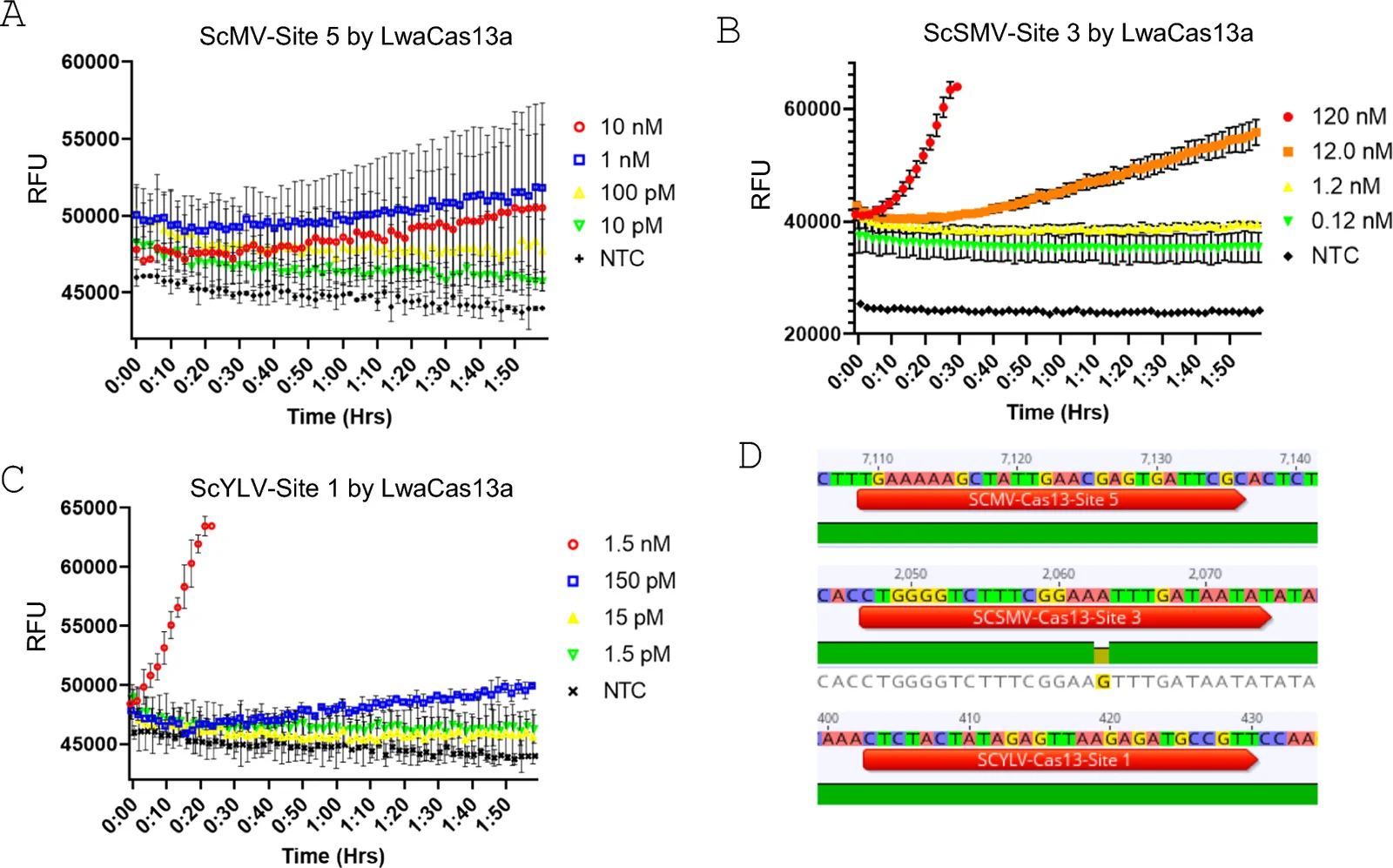
Best performing test runs of selected target sites for each virus. The limit of detection (LOD) was established for each top-performing target site for each virus: Site 5 for ScMV (**A**), Site 3 for ScSMV (**B**), and Site 1 for ScYLV (**C**). These were tested by making log dilutions of our IVT target ssRNA containing “mock target sites”. Differences in concentrations between tests reflect the relative IVT efficiency for each synthesized sequence. Overall, our assay captured a minimum target RNA concentration of ~1.5 pM. (**D**) Sequences of our best-performing crRNA design for each virus. These alignments and crRNA designs were generated with Geneious Prime software

Our assay used the type VI CRISPR-Cas protein LwaCas13a. In our design, we aimed to achieve multiplexed detection of different viruses by multiplexing corresponding crRNAs for the viruses of interest, and to set up these assays easily in a plate (Fig. 3). To develop this assay with the crRNAs and pseudo-virus sequences, we first aimed to determine the limit of detection (LOD): the lowest concentration of pathogenic RNA that LwaCas13a (with a 1:1 molar ratio of the crRNA) could detect and still produce a detectable fluorescent signal. We performed a serial log dilution of our IVT-synthesized RNA to determine the lowest concentration that still produced a detectable signal compared with the NTC. We used our crRNA designs for each of the three viruses at different target sites as well (Fig. 4A-D; **Suppl. Figure 1–3**). The LOD varied between target sites, but overall, the lowest concentration of LwaCas13a that still achieved detection was ~1.5 pM (Fig. 4C). With this assay, we were able to validate and select the best-performing target site for each virus to further develop our assays. Those target sites were Site 5 on ScMV, Site 3 on ScSMV, and Site 1 for ScYLV.

### Multiplexing Target Sites/crRNAs for Simultaneous Detection and Genotyping

**Fig. 5:**
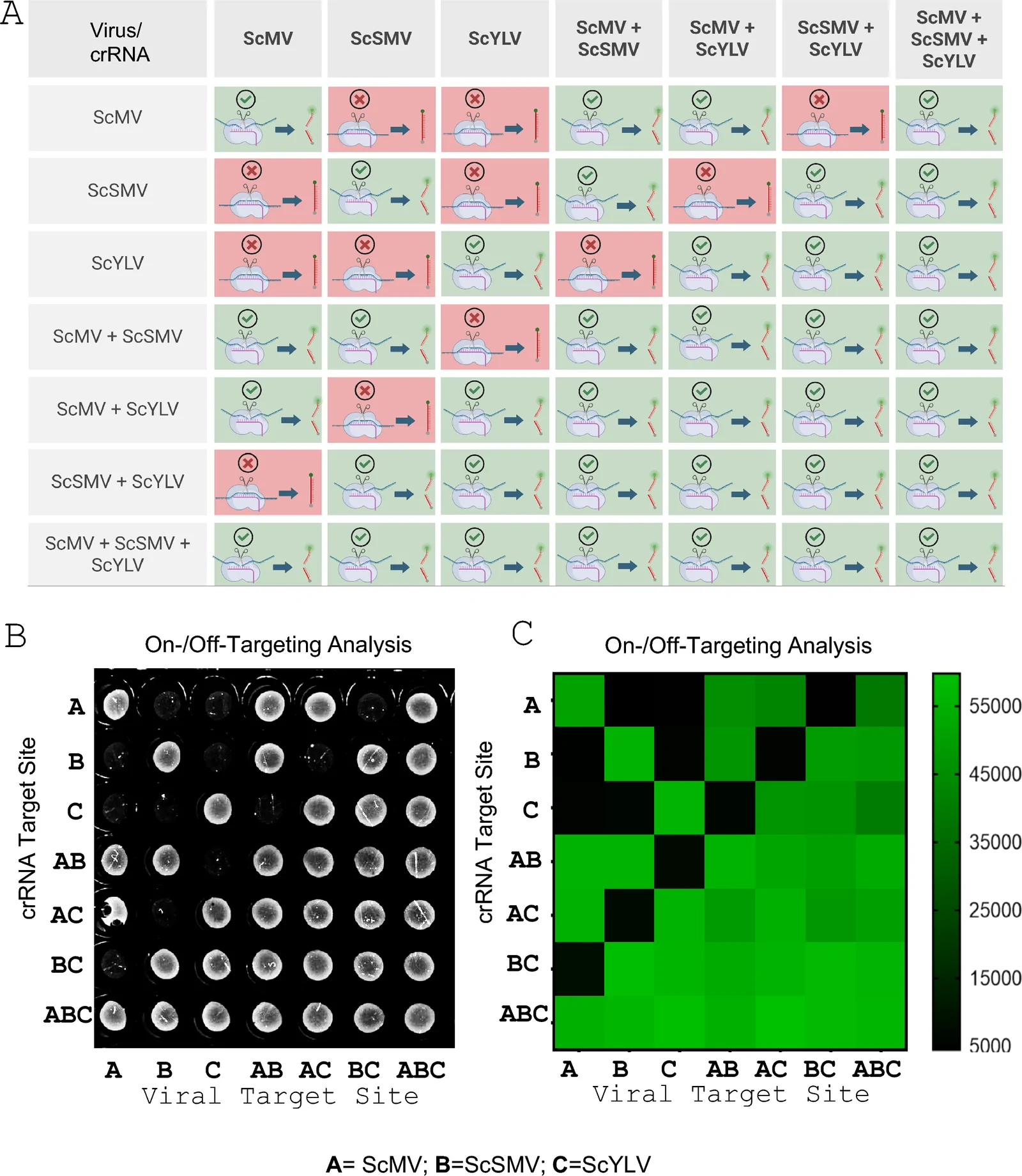
Test for multiplexed virus sequence detection. (**A**) Design of the validation test for targeting specificity. A green “√” represents an expected positive detection, while a red “⮾” represents a well not suspected to have a positive detection. Horizontally: ssRNA pseudo-viruses are synthesized via an IVT kit, each containing target sites for the labeled virus in high concentration. Vertically: A ribonucleoprotein (RNP) complex was created via the addition of LwaCas13a and the labeled crRNA designed to target that particular virus. (**B**) After running for 15 min, the plate was placed in a gel doc reader to observe the fluorescence. (**C**) Quantitative readings collected from the fluorescent plate reader. Wells with the correct combination of virus spiked in the well, and crRNA had RFU values ranging from 38,974 to 59,782. Wells lacking the correct combination had much lower RFU values, ranging from 4458 to 8147

To ensure assay robustness, we next verified that the selected target sites did not cross-react. We tested every combination of synthesized viral targets and crRNAs to assess on-/off-targeting for each target site. All other reagents in each well were identical. A high concentration of each pseudo-virus target RNA was used to saturate each well with target sites. The same test parameters and machine settings were used for this assay as were used for the LOD tests. Based on our design, we predicted detection outcomes for positive and negative samples (Fig. 5A). Our experimental results exactly confirmed this prediction (Fig. 5B). Interestingly, after about 15–20 min of running the detection reaction, the fluorescent signal was so strong that it was visible under a gel doc reader, most commonly used to observe bands on a gel electrophoresis (Fig. 5B). This is also in agreement with the raw values of Relative Fluorescent Units (RFU) detected by the fluorescent microplate reader (Fig. 5C). The most promising from these experiments was a demonstration that our system is highly specific to each virus, with no cross-reaction between them. We tested each virus mixture with each crRNA combination in a matrix configuration, and observed positive detection only when the viral target matched the crRNA targeting that virus. This demonstrated the specificity of a genotyping strategy for these 3 viruses. A sugarcane sample suspected of being positive, or confirming healthy material, for any of these viruses could be tested with each crRNA design in one assay for rapid and robust results if any of these viruses are present in the sugarcane tissue, as we proposed (Fig. 3).

### Detection assay validation on real infected plant samples

**Fig. 6:**
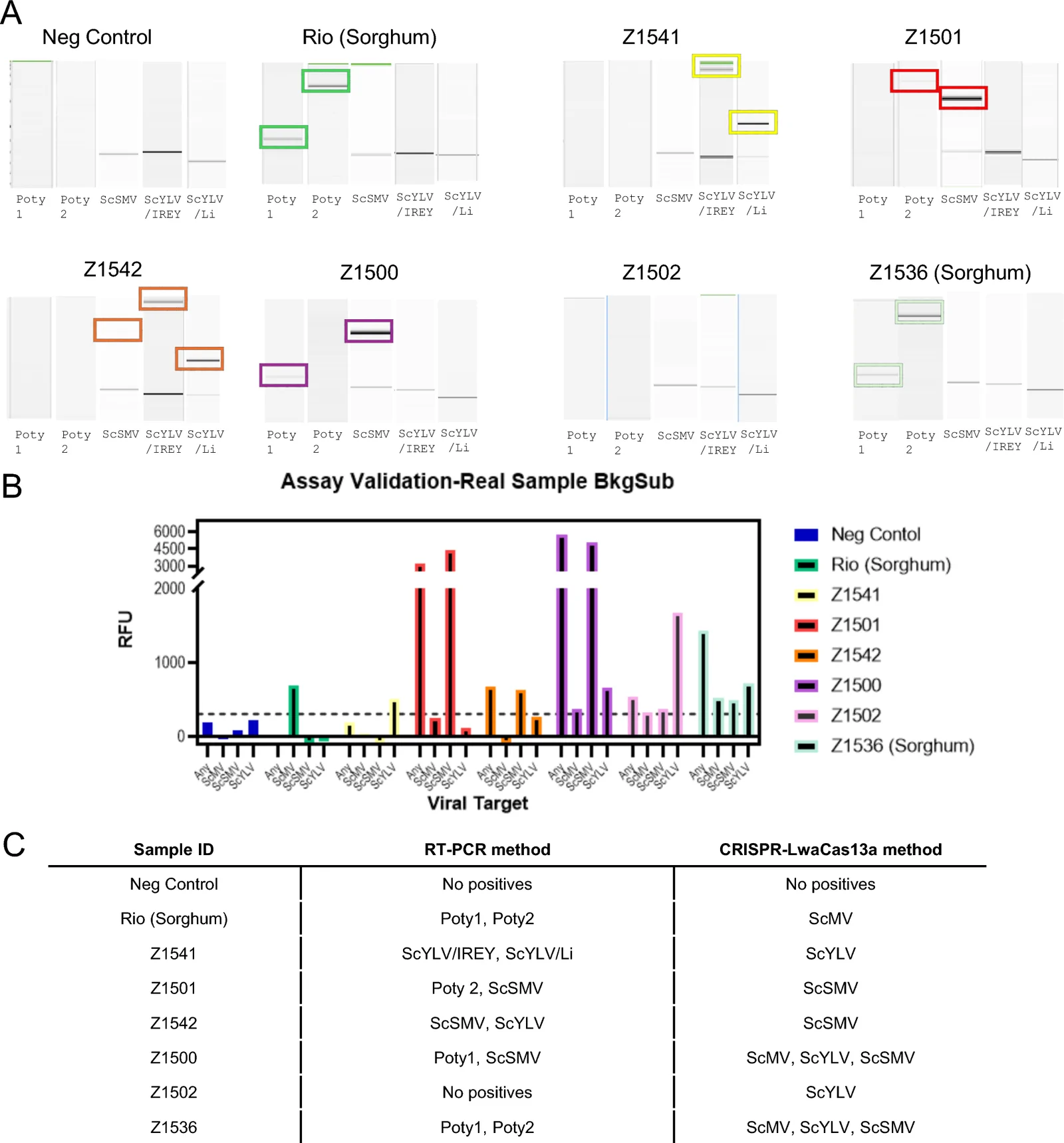
Detection of target viruses from real plant samples. (**A**) Detection of viruses with five different primer sets using the conventional RT-PCR assays. In these assays, ScSMV, ScYLV/IREY, and ScYLV/Li also used a second set of primers for Nad5, an internal control of the host plant. They present a lower band, and virus positivity is determined by a second band higher than the Nad5 control. Positive bands are highlighted with red rectangles. (**B**) CRISPR-LwaCas13a detection of the three quarantine-significant viruses. Each value is background-subtracted against the average RFU for blank wells and NTC (6902.167 RFU). This test was performed simultaneously with a confirmed healthy sugarcane plant sample as our “Neg Control”. A dashed line is drawn across to represent three standard deviations above the mean of values for the negative control. (**C**) A table that summarizes the detection results from the RT-PCR method and the LwaCas13a method

While we verified that our crRNAs were specific to each virus with no cross-reactivity to other viral targets, we performed these assays on highly concentrated, synthesized ssRNA containing our target sites. Next, we tested the assay's robustness on extractions directly from verified infected sugarcane and sorghum samples (another plant host). We worked closely with USDA-APHIS, Plant Germplasm Quarantine Program (Laurel, MD, USA), to test at their quarantine facility on the infected plants they keep on-site. We extracted RNA from these plants using the same strategy used by USDA-APHIS PGQP diagnosticians. First, we used the conventional RT-PCR assays to screen the selected plant tissues. Different viruses were detected from some potentially infected plants using these established assays, whereas no positive bands were amplified from the negative, healthy plants (Fig. 6A).

For LwaCas13a-based detection, we observed that our system not only detected sugarcane viruses in real samples but also identified which virus was causing the infections in the tissues (Fig. 6B; Suppl. Figure 4; Suppl. Table 2). Any sample’s fluorescence that was greater than one standard deviation above the highest value produced by the Negative Control (healthy sugarcane tissue) was considered positive. Based on the peaks, we could see which sugarcane viruses were present in each sugarcane and sorghum tissue sample. The LwaCas13a-based assay and the RT-PCR-based assay were consistent in many samples (Fig. 6A; 6B). However, inconsistent results were found for some samples.

For example, ‘Rio’ is sorghum tissue that was found to be positive for Poty1 and Poty2 viruses in the conventional assay, yet the CRISPR assay was able to identify that ‘Rio’ is most positive for ScMV (Fig. 6A; 6B). This was demonstrated by RT-PCR, which showed results similar to the CRISPR assay. This is the case specifically for the Poty1 and Poty2 detection protocols, which only detect the presence of a virus in the *Potyvirus* genus but do not actually identify which specific virus in the genus is present without sequencing, an additional diagnostic step not required by our CRISPR assay. The other sorghum tissue tested was ‘Z1536’, which was positive for both Poty1 and Poty2 protocols by RT-PCR (Fig. 6A) but did not produce a positive result for any of the other protocols. Our CRISPR-based assay did, however, detect a signal above our threshold for all three viruses being screened in the ‘Z1536’ tissue sample (Fig. 6B).

For the sugarcane sample ‘Z1501’, there was a very weak band for the Poty2 primer set, but not in the Poty1 primers. There was, however, a very strong band for ScSMV. This suggests that the sample does contain ScSMV, yet it is peculiar that the band was quite weak for the Poty2 protocol and undetectable for the Poty1 protocol (Fig. 6A). This may be due to some level of weak cross-reactivity between the Poty2 primer set and ScSMV. In the CRISPR-based assay, ‘Z1501’ did, however, produce a large signal for the crRNA targeting ScSMV. There was some signal for the other viruses, yet not enough signal was produced for us to determine these as true positives (Fig. 6B).

The sugarcane sample ‘Z1542’ gave interesting results. Based on the conventional RT-PCR assay (Fig. 6A), the sample showed a weak band for ScSMV and two distinct bands for each ScYLV test, yet the CRISPR assay produced a strong positive signal only for ScSMV (Fig. 6B). Some signal appeared to be detected for ScYLV in the same sample, but it was still below our threshold for a positive result. Sugarcane ‘Z1502’ did not appear to have any detectable bands for all the viruses in the RT-PCR tests (Fig. 6A). The CRISPR assay was able to detect the presence of ScYLV in the tissue (Fig. 6B).

**Fig. 7:**
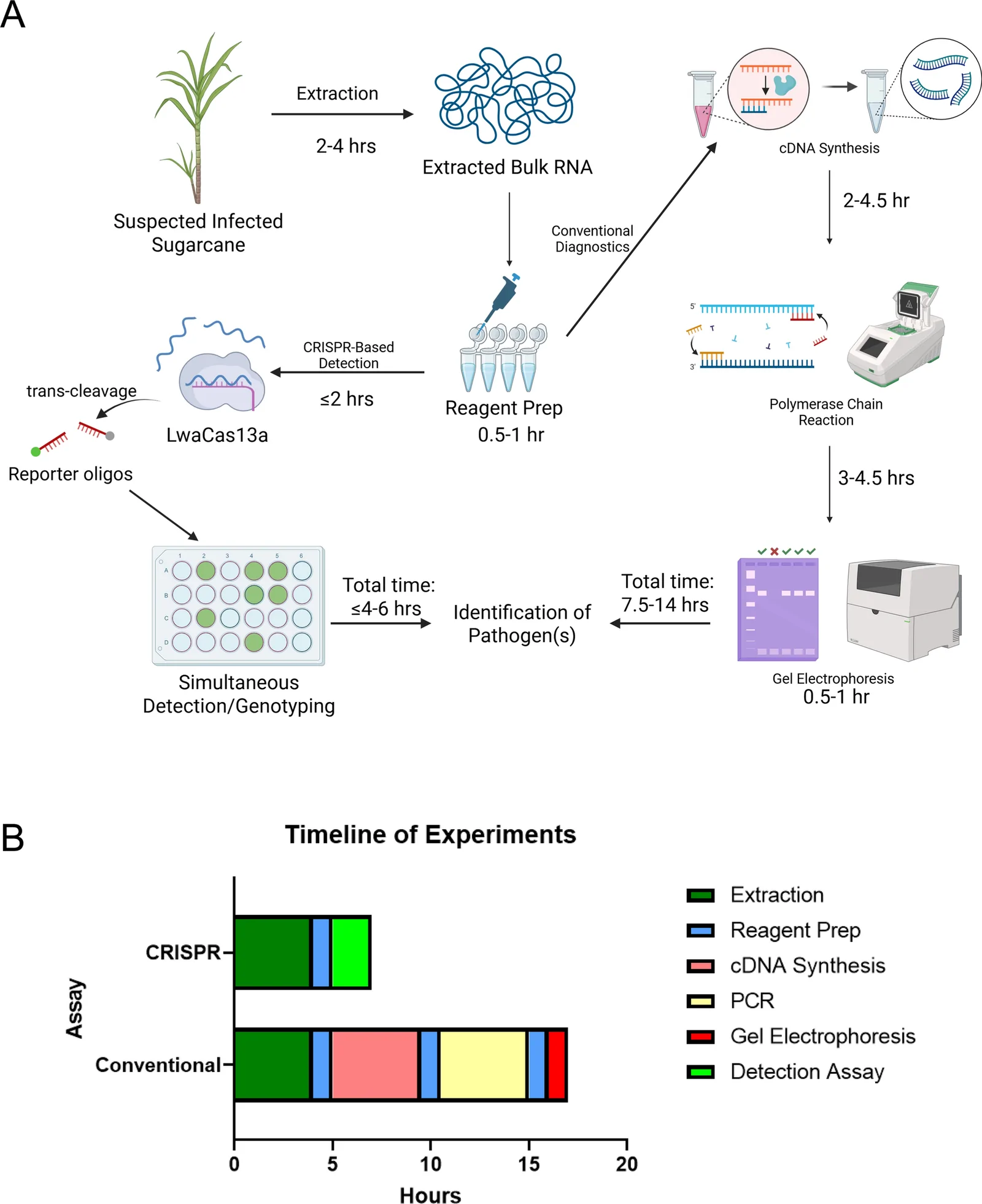
Comparison of the conventional and CRISPR-based detection methods. (**A**) Comparison of different steps in the conventional (RT-PCR-based) and the CRISPR-Cas13-based methods. The assay with LwaCas13a simultaneously detects and identifies three quarantine-significant sugarcane viruses directly from extraction without a pre-amplification step. The CRISPR test runs for 2 h or less, compared to conventional diagnostic methods that require more than double the time. The diagram was generated with BioRender. (**B**) A graph demonstrating how much less time is needed to test all three quarantine-significant viruses simultaneously compared to performing the conventional assay for each virus.

Overall, the results show that our CRISPR-based detection platform can detect viruses directly from bulk RNA-extracted material. It does not require a pre-amplification step and can identify which specific virus is present in a single protocol (Fig. 7).

## Discussion

Sugarcane is the most highly cultivated crop in many sub-tropical/tropical climates(Xu et al. 2021; Dinesh Babu et al. 2022). Some countries’ economies rely heavily on sugarcane production and trade since it is so highly cultivated (Kumar et al. 2018; Dinesh Babu et al. 2022). While a great deal of research has been focused on increasing yield, our approach was to protect the crops already being grown by improving our detection capacity to rapidly find virus-infected tissue. Virus outbreaks, particularly by the three quarantine-relevant viruses tested here, would cause significant damage to crop yield if not detected early and left unchecked (Vijai Singh 2003; Viswanathan and Balamuralikrishnan 2005; Holkar et al. 2020).

The use of CRISPR-Cas13 for the detection of plant viruses without a preamplification step was previously demonstrated (Hak, et al. 2025; Marques, et al. 2022). In this study, we attempted to develop a CRISPR-Cas13-based assay for detection of quarantine-significant RNA viruses. Our LOD of ~1.5 pM is about a 10-fold improvement over the recent report that used a CRISPR-Cas13-based method for rapid detection of plant viruses (Hak, et al. 2025). However, Cas13 enzyme kinetics limit its intrinsic detection sensitivity in an amplification-free application (Huyke, et al. 2022). The sensitivity of our method may not be high enough to detect plant tissues with light infection. For early detection of low virus titers, it may be necessary to use RT-PCR or add a preamplification step, as used in SHERLOCK (Kellner, et al. 2019). Also, it would be interesting to compare this CRISPR-Cas13-based detection method with a high-throughput sequencing (HTS)-based method, as the latter is also used for virus identification in plants (Al Rwahnih, et al. 2015; Soltani, et al. 2021; Vigne, et al. 2018).

In the conventional RT-PCR-based methods, 5 separate protocols are needed for different viruses. Each protocol can be done in parallel, but many more steps are still needed to test all samples. In contrast, our CRISPR-Cas13-based assay can screen for each pathogen simultaneously by multiplexing and by including multiple specific assays on the same microplate. Each sample can be tested for any of the viruses we are screening for in the ‘Any’ well and then can be tested for each virus individually in the other wells, all simultaneously. This allows detection and identification of which virus might be infecting a sugarcane crop in a single test. Our CRISPR-based detection test is sensitive enough to avoid pre-amplification steps while remaining specific enough to avoid cross-reactivity with other viruses. The assay was validated with infected plants and was able to determine which pathogen each plant contained in four hours, or even less for high-titer infections (Fig. 7A). In comparison, the conventional RT-PCR methods would require much more time (Fig. 7B).

Our developed strategy will not only save time but also detect the virus in a single protocol, greatly simplifying the testing procedure. What might take a skilled laboratory technician a day or two (assuming an 8-h workday and smooth experiments) to test even one sample will instead take less than a day’s work to test for all viruses simultaneously (Fig. 7A**; 7B**). It should be noted that to test for all three viruses, there were five separate RT-PCR protocols to use for screening. Figure 7 only provides a comparison of performing one RT-PCR protocol versus one run of our CRISPR-based assay. This could increase the time greatly beyond our estimates since we were only accounting for the time estimates of one of these protocols. Since it is common that these viruses may come with co-infections, establishing an assay that can determine which viruses are present within the sample is a powerful tool for diagnosticians working with these diseases. The multiplexed CRISPR assay, which can be run on one plate, simultaneously tests for “Any” of the three viruses and identifies which specific one(s) are present, is a much faster and more efficient diagnostic workflow for molecular diagnostics of sugarcane viruses than current conventional testing for the same pathogens.

Another benefit of the CRISPR-based strategy is its ability to better quantify virus titer in tissue because it produces a stronger signal in fluorescent microplate readings. The conventional RT-PCR assay is a qualitative presence/absence assay, and it is harder to determine titer in plant tissue. Another advantage of our strategy is that working directly from extracted tissue provides faster turnaround and is more direct by eliminating additional steps. Testing with the extracted material in a single step following the extraction greatly reduces chances for contamination, which can occur in the conventional RT-PCR workflow during cDNA preparation or while setting up any of the five individual PCRs. This is an essential consideration when working with diagnostic assays to reduce as many opportunities for contamination as possible.

We observed some inconsistencies when comparing the RT-PCR method with our CRISPR-Cas13 method. Because this CRISPR assay uses ssRNA reporters and is performed at 37 °C for 2 h, these reporters may naturally degrade during the reaction, causing false positives at later time points. Our threshold strategy resolved this by determining samples as positive when their RFU result was greater than three standard deviations above the mean of background-subtracted negative control RFU values (background subtraction came from the average RFU of the blank well and NTC RFUs subtracted from each well).

The analytical sensitivity of the RT-PCR assays for Poty1, Poty2, ScSMV, and ScYLV (IREY and Li) is not well characterized, which may explain the differences between the CRISPR and PCR results. It is possible that a relatively weakly positive result in the CRISPR assay, e.g., ScSMV and ScYLV CRISPR detection in ‘Z1536’, was not detected in the corresponding RT-PCR assays due to low virus titer. Viral titer can also vary seasonally and within/across plant tissue, affecting downstream RNA concentration and quality. Also, the RT-PCR method relies on cDNA synthesis first, then amplification of target regions. This two-step process can add more error or variability to the assay, while the CRISPR-based assay can interact directly with the extracted material. Furthermore, rapid virus evolution may also account for some of the discrepancies we observed between the two detection methods. That’s why there are two separate assays to try to capture ScYLV in samples (Holkar et al. 2020; Malapi-Wight et al. 2021; Sood et al. 2021). Mutations at the primer sites may disrupt the RT-PCR assays' ability to correctly detect a positive result, even when the virus is present. This could be due to the low titer of virus in the tissue during the time of extraction. It has been documented that there is significant diversity among ScYLV, which is why two primer sets are used in the RT-PCR protocols (Rott et al. 2023). This can account for variability in results between isolates and time points, eventually reducing reliability. This did not seem to be a problem for the target sites selected for our CRISPR-based approach, probably because the CRISPR targets were recently designed in conserved regions using up-to-date sequence data.

Of course, our target sites could also lose reliability, but a CRISPR-based assay needs fewer regions to work than a conventional PCR assay. Standard PCR requires at least two target sites (more sites are required for the probe(s) in RT-qPCR or when multiplexing) to be complementary to the forward/reverse primer set to work, given the test is performed with the correct cycling conditions. However, evaluating positive or negative PCR results usually requires an additional test, most often gel electrophoresis, to observe bands of the correct/expected amplicon size (s). As with any test, it still has flaws because it does not account for mutations acquired at the amplification sites or large deletions or insertions that could drastically change the expected band size. While our study was constrained by the limited number of samples infected with these sugarcane viruses, our established CRISPR-Cas13-based protocol provides a strong starting point for testing more field samples in the future. Testing more samples would allow for more rigorous statistical analysis, which may help benchmark CRISPR-Cas13-based detection as a reliable alternative to the RT-PCR-based assay.

CRISPR-based detection, on the other hand, would only require one target site to be complementary to the crRNA spacer region for the test to be viable. Redesign is straightforward since only one region requires a new design, and LwaCas13a has an additional advantage: it does not require a PAM (protospacer adjacent motif) site (Abudayyeh et al. 2017a, b; Cox et al. 2017). Cas proteins like Cas9 and Cas12 require a PAM site which makes target selection more stringent (Anders et al. 2014; Kleinstiver et al. 2015; Yan et al. 2019). When redesigning for standard PCR, much more must be considered, including non-optimal annealing temperatures, primer-dimerization, and an appropriate elongation period for amplicon length, among other factors. For CRISPR-based detection, the requirements for testing mostly include a target-specific crRNA design and an appropriate method for capturing the signal from trans-cleavage activity. We envision further improving our plant virus detection assay with kinetic barcoding in multiplexed detection, new design strategies to improve specificity, and improved trans-cleavage activity (Molina Vargas et al. 2023; Yang et al. 2024; Son et al. 2026). These improvements would support the adoption of CRISPR-Cas13 for detecting different RNA viruses to improve crop protection and productivity.

## Conclusion

Here, we developed a plant RNA virus detection method using CRISPR-Cas-LwaCas13a to screen for three sugarcane viruses of great significance to global agricultural systems. We used this strategy to detect sugarcane viruses in infected sugarcane and sorghum tissue and compared it with established conventional RT-PCR techniques for the same viruses. The developed assay can be performed more rapidly, requires fewer steps, detects and identifies viruses in a single test, and can detect traces of virus that RT-PCR could not capture in some cases. We tested the CRISPR-Cas13 assay directly with bulk-extracted RNA from infected tissue, and it required no additional prep steps. As a result, we strengthened diagnostic capacity for rapid sugarcane virus screening beyond what was available before. We envision expanding this technology to screen for many more viruses, pathogens, or molecular markers simultaneously, giving researchers a panel of information from a single assay. Equipped with cold storage and an appropriate plate reader, this work could also facilitate the development of rapid, field-deployable virus diagnostic methods, which would be especially valuable for early detection and containment during severe disease outbreaks.

## Supplementary Information

Below is the link to the electronic supplementary material.

Supplementary file1 (DOCX 21 KB)

Supplementary file2 (PPTX 15157 KB)

## Data Availability

All data supporting the findings of this study are available within the paper and its Supplementary Information.
