# Supplementary material for: CRISPR-Cas13-based amplification-free detection of three quarantine-significant sugarcane viruses
Source: Plant Cell Rep. 2026 Sep 26;45(10):307. doi: 10.1007/s00299-026-03994-4 (PMC13615938; doi:10.1007/s00299-026-03994-4)
Supplement: Supplementary file 1 [file 299_2026_3994_MOESM1_ESM.docx]

|  | Target Sequence (5'->3') | Target RNA  (5'->3') | RNA Reverse Compliment –Spacer (RNA)  (5'->3') | LwaCas13a crRNA Sequence (5’->3’) |
| --- | --- | --- | --- | --- |
| SCMV-Site 1 | AAGTTATCGATTGGAAAGCTAGTGATTC | AAGUUAUCGAUUGGAAAGCUAGUGAUUC | GAAUCACUAGCUUUCCAAUCGAUAACUU | GACUACCCCAAAAACGAAGGGGACUAAAACGGAAUCACUAGCUUUCCAAUCGAUAACU |
| SCMV-Site 2 | ATTACAAACCATTCTTAACCGTGAGAAA | AUUACAAACCAUUCUUAACCGUGAGAAA | UUUCUCACGGUUAAGAAUGGUUUGUAAU | GACUACCCCAAAAACGAAGGGGACUAAAACUUUCUCACGGUUAAGAAUGGUUUGUAAU |
| SCMV-Site 3 | ACAAATCAAGACTTACCTGCAGACACAT | ACAAAUCAAGACUUACCUGCAGACACAU | AUGUGUCUGCAGGUAAGUCUUGAUUUGU | GACUACCCCAAAAACGAAGGGGACUAAAACAUGUGUCUGCAGGUAAGUCUUGAUUUGU |
| SCMV-Site 4 | CCAAAGGTGAACTTTCACAATTGGTTGA | CCAAAGGUGAACUUUCACAAUUGGUUGA | UCAACCAAUUGUGAAAGUUCACCUUUGG | GACUACCCCAAAAACGAAGGGGACUAAAACUCAACCAAUUGUGAAAGUUCACCUUUGG |
| SCMV-Site 5* | TGAAAAAGCTATTGAACGAGTGATTCGC | UGAAAAAGCUAUUGAACGAGUGAUUCGC | GCGAAUCACUCGUUCAAUAGCUUUUUCA | GACUACCCCAAAAACGAAGGGGACUAAAACGCGAAUCACUCGUUCAAUAGCUUUUUCA |
| ScSMV-Site 1 | ACAAACAAGCATACATCTCAACCTCTCT | ACAAACAAGCAUACAUCUCAACCUCUCU | AGAGAGGUUGAGAUGUAUGCUUGUUUGU | GACUACCCCAAAAACGAAGGGGACUAAAACAGAGAGGUUGAGAUGUAUGCUUGUUUGU |
| ScSMV-Site 2 | CATCATGACATCATTCCAGCTACTGAAA | CAUCAUGACAUCAUUCCAGCUACUGAAA | UUUCAGUAGCUGGAAUGAUGUCAUGAUG | GACUACCCCAAAAACGAAGGGGACUAAAACUUUCAGUAGCUGGAAUGAUGUCAUGAUG |
| ScSMV-Site 3* | CTGGGGTCTTTCGGAAATTTGATAATAT | CUGGGGUCUUUCGGAAAUUUGAUAAUAU | AUAUUAUCAAAUUUCCGAAAGACCCCAG | GACUACCCCAAAAACGAAGGGGACUAAAACAUAUUAUCAAAUUUCCGAAAGACCCCAG |
| ScSMV-Site 4 | TACTATCAAACAATTGATTTTGAAATTC | UACUAUCAAACAAUUGAUUUUGAAAUUC | GAAUUUCAAAAUCAAUUGUUUGAUAGUA | GACUACCCCAAAAACGAAGGGGACUAAAACGAAUUUCAAAAUCAAUUGUUUGAUAGUA |
| ScSMV-Site 5 | ATACTTGGAACAGAGCTTTTATCATCGC | AUACUUGGAACAGAGCUUUUAUCAUCGC | GCGAUGAUAAAAGCUCUGUUCCAAGUAU | GACUACCCCAAAAACGAAGGGGACUAAAACGCGAUGAUAAAAGCUCUGUUCCAAGUAU |
| SCYLV-Site 1* | CTCTACTATAGAGTTAAGAGATGCCGTT | CUCUACUAUAGAGUUAAGAGAUGCCGUU | AACGGCAUCUCUUAACUCUAUAGUAGAG | GACUACCCCAAAAACGAAGGGGACUAAAACAACGGCAUCUCUUAACUCUAUAGUAGAG |
| SCLYV-Site 2 | CCCACCGACTGTTCCGGTTTTGACTGGT | CCCACCGACUGUUCCGGUUUUGACUGGU | ACCAGUCAAAACCGGAACAGUCGGUGGG | GACUACCCCAAAAACGAAGGGGACUAAAACACCAGUCAAAACCGGAACAGUCGGUGGG |
| SCLYV-Site 3 | CATGATCCACAGCTAGTTGCTACCCTCC | CAUGAUCCACAGCUAGUUGCUACCCUCC | GGAGGGUAGCAACUAGCUGUGGAUCAUG | GACUACCCCAAAAACGAAGGGGACUAAAACGGAGGGUAGCAACUAGCUGUGGAUCAUG |
| SCYLV-Site 4 | TCCCGGCCCCCTTCATCAGGGGGAAAGA | UCCCGGCCCCCUUCAUCAGGGGGAAAGA | UCUUUCCCCCUGAUGAAGGGGGCCGGGA | GACUACCCCAAAAACGAAGGGGACUAAAACUCUUUCCCCCUGAUGAAGGGGGCCGGGA |
| SCLYV-Site 5 | ACAACCAGCGAGATAAACCTGTGGTGGA | ACAACCAGCGAGAUAAACCUGUGGUGGA | UCCACCACAGGUUUAUCUCGCUGGUUGU | GACUACCCCAAAAACGAAGGGGACUAAAACUCCACCACAGGUUUAUCUCGCUGGUUGU |

**Supplementary Table 1. Sequence data for target sequences in ScMV, ScSMV, and ScYLV.** Also included are the designed crRNA sequences used to target these sequences. The sequences that were used in the actual assay are annotated with a “*”.

*Non-Background-subtracted RFU Values (Average of 3 replicants)*

| **Sample Name** | **All Viruses** | **ScMV** | **ScSMV** | **ScYLV** |
| --- | --- | --- | --- | --- |
| Negative (Healthy) | 7277.33 | 7042.67 | 7164.33 | 7326.667 |
| *Rio* | 7167.33 | 7830.00 | 6915.33 | 6982.33 |
| *Z1541* | 7315.33 | 7219.00 | 6926.67 | 7641.667 |
| *Z1501* | 12957.00 | 7380.33 | 14407.67 | 7273 |
| *Z1542* | 7978.00 | 7010.00 | 7835.00 | 7435.667 |
| *Z1500* | 12902.00 | 7526.33 | 12200.33 | 7813.333333 |
| *Z1502* | 7683.67 | 7476.67 | 7514.33 | 8819 |
| *Z1536* | 8582.67 | 7675.00 | 7645.00 | 7868.666667 |
| *NTC** | 7116.666667 | 7093.333333 | 7150 | 7090 |
| *Blank** | 6983 | 7159 | 7113 | 6984 |
| ** -omitted from final figures but used for background-subtracted normalization* | | | | |
|  |  |  |  |  |
| Background-subtracted RFU Values (Average of 3 replicants) (BkgSUB RFU= 7086.125) | | | | |
| **Sample Name** | **All Viruses** | **ScMV** | **ScSMV** | **ScYLV** |
| Negative (Healthy) | 191.20 | -43.46 | 78.20 | 240.54 |
| *Rio* | 81.21 | 743.87 | -170.80 | -103.80 |
| *Z1541* | 229.20 | 132.87 | -159.46 | 555.54 |
| *Z1501* | 5870.87 | 294.20 | 7321.54 | 186.87 |
| *Z1542* | 891.87 | -76.13 | 748.87 | 349.54 |
| *Z1500* | 5815.87 | 440.21 | 5114.21 | 727.21 |
| *Z1502* | 597.54 | 390.54 | 428.21 | 1732.87 |
| *Z1536* | 1496.54 | 588.87 | 558.87 | 782.54 |

**Supplementary Table 2. Raw RFU reads of the microplate reader for virus detection from plant materials.** Final RFU values after a 2-hour runtime at 37° C.
